# Excess mortality associated with mental illness in people living with HIV in Cape Town, South Africa: a cohort study using linked electronic health records

**DOI:** 10.1016/S2214-109X(20)30279-5

**Published:** 2020-10

**Authors:** Andreas D Haas, Yann Ruffieux, Leigh Luella van den Heuvel, Crick Lund, Andrew Boulle, Jonathan Euvrard, Catherine Orrell, Hans W Prozesky, Nicki Tiffin, Kathryn L Lovero, Mpho Tlali, Mary-Ann Davies, Milton L Wainberg, IeDEA Southern Africa collaboration

**Affiliations:** Institute of Social and Preventive Medicine, University of Bern, Bern, Switzerland (A D Haas PhD, Y Ruffieux MSc); Department of Psychiatry, Faculty of Medicine and Health Sciences (L L van den Heuvel MMed) and Division of Infectious Diseases, Department of Medicine, Tygerberg Academic Hospital (H W Prozesky MMed), University of Stellenbosch, Cape Town, South Africa; Alan J Flisher Centre for Public Mental Health, Department of Psychiatry and Mental Health (Prof C Lund PhD), Centre for Infectious Disease Epidemiology and Research (Prof A Boulle PhD, J Euvrard MPH, N Tiffin PhD, M Tlali MBChB, Prof M-A Davies PhD), Institute of Infectious Disease and Molecular Medicine (C Orrell MBChB), Wellcome Centre for Infectious Disease Research in Africa (N Tiffin), and Division of Computational Biology (N Tiffin), University of Cape Town, Cape Town, South Africa; King’s Global Health Institute, Centre for Global Mental Health, Health Service and Population Research Department, Institute of Psychiatry, Psychology and Neuroscience, King’s College London, London, UK (Prof C Lund); Western Cape Provincial Department of Health, Cape Town, South Africa (Prof A Boulle); and Department of Psychiatry and New York State Psychiatric Institute, Columbia University, New York, NY, USA (K L Lovero, Prof M L Wainberg MD)

## Abstract

**Background:**

Mental disorders can adversely affect HIV treatment outcomes and survival. Data are scarce on premature deaths in people with mental disorders in HIV-positive populations, particularly in low-income and middle-income countries. In this study, we quantified excess mortality associated with mental disorders in HIV-positive people in South Africa, adjusting for HIV treatment outcomes.

**Methods:**

For this cohort study, we analysed routinely collected data on HIV-positive adults receiving antiretroviral therapy (ART) in Cape Town, South Africa between Jan 1, 2004, to Dec 31, 2017. Data from three ART programmes were linked with routine medical records on mental health treatment from Jan 1, 2010, to Dec 31, 2017, and mortality surveillance data from the South African National Population Register up to Dec 31, 2017. People living with HIV aged 15 years or older who initiated ART at a programme site were eligible for analysis. We followed up patients from ART initiation or Jan 1, 2010, whichever occurred later, to transfer, death, or Dec 31, 2017. Patients were considered as having a history of mental illness if they had ever received psychiatric medication or been hospitalised for a mental disorder. We calculated adjusted hazard ratios (aHRs) with 95% CIs for associations between history of mental illness, mortality, and HIV treatment outcomes (retention in care with viral load suppression [VLS; viral load <1000 copies per mL], retention in care with non-suppressed viral load [NVL; viral load ≥1000 copies per mL], and loss to follow-up [LTFU; >180 days late for a clinic visit at closure of the database]) using Cox proportional hazard regression and multistate models.

**Results:**

58 664 patients were followed up for a median of 4·3 years (IQR 2·1–6·4), 2927 (5·0%) of whom had a history of mental illness. After adjustment for age, sex, treatment programme, and year of ART initiation, history of mental illness was associated with increased risk of mortality from all causes (aHR 2·98 [95% CI 2·69–3·30]), natural causes (3·00 [2·69–3·36]), and unnatural causes (2·10 [1·27–3·49]), compared with no history of mental illness. Risk of all-cause mortality in people with a history of mental illness remained increased in multivariable analysis adjusted for age, sex, treatment programme, year of ART initiation, CD4 count and WHO clinical stage at ART initiation, retention in HIV care with or without VLS, and LTFU (2·73 [2·46–3·02]). In our multistate model, adjusted for age, sex, year of ART initiation, cumulative time with NVL, and WHO clinical stage and CD4 cell count at ART initiation, rates of excess all-cause mortality in people with history of mental illness were greatest in patients retained in care with VLS (aHR 3·43 [95% CI 2·83–4·15]), followed by patients retained in care with NVL (2·74 [2·32–3·24]), and smallest in those LTFU (2·12 [1·78–2·53]). History of mental illness was also associated with increased risk of HIV viral rebound (transitioning from VLS to NVL; 1·50 [1·32–1·69]) and LTFU in people with VLS (1·19 [1·06–1·34]).

**Interpretation:**

Mental illness was associated with substantial excess mortality in HIV-positive adults in Cape Town. Excess mortality among people with a history of mental illness occurred independently of HIV treatment success. Interventions to reduce excess mortality should address the complex physical and mental health-care needs of people living with HIV and mental illness.

**Funding:**

National Institutes of Health, Swiss National Science Foundation, South African Medical Research Council.

## Introduction

In 2017, mental illness was a leading cause of disease burden in Africa. The South African Stress and Health study, which collected national population data between 2003, and 2004, showed that mental disorders affected one in three adults in South Africa during their lifetime.^1^ Anxiety, depression, and substance use disorders were the most common mental disorders, each affecting more than 10% of the population at least once during life.^1^ Severe mental disorders including schizophrenia, psychosis, and bipolar disorder are not as common, usually occurring in less than 2% of the population.

People with mental disorders have higher mortality than the general population. Excess mortality in mental disorders has been well documented for high-income countries.^2^ A meta-analysis of 148 studies found that the pooled relative risk of all-cause mortality in people with mental disorders was two times higher than that in individuals without a mental disorder or the general population.^2^ However, most studies included in the meta-analysis were from Europe or North America. Only six studies were from low-income and middle-income countries, of which only two were from Africa. Both studies were done in Ethiopia and showed standardised mortality ratios of about 4 in people with major depression and 6 in people with schizophrenia.^3,4^

Although mental disorders are prevalent in people living with HIV^5^ and associated with poor adherence to life-saving antiretroviral therapy,^6^ data are scarce on premature deaths in people with mental disorders in HIV-positive populations, and mostly restricted to high-income countries.^7–9^ One study in Tanzania showed that depressive symptoms at ART initiation were associated with a two times higher risk of mortality in women living with HIV.^10^ In the current study, we aimed to evaluate excess mortality associated with mental disorders in people living with HIV. We quantified excess mortality in people with a history of mental illness (ie, people who had ever been hospitalised for a mental disorder or received psychiatric medications) among HIV-positive adults who had initiated ART in three HIV programmes in Cape Town, South Africa, and examined associations between a history of mental illness and HIV treatment outcomes.

## Methods

### Study design

In this cohort study, we analysed routinely collected data on HIV-positive adults receiving ART in South Africa. We linked data from three ART programmes in Cape Town to data on mental health treatment in Western Cape province and national mortality surveillance data. ART programme data covering the period from Jan 1, 2004, to Dec 31, 2017, were provided by the International Epidemiology Database to Evaluate AIDS (IeDEA) Southern Africa collaboration.^11^ Mental health records covering the period from Jan 1, 2010 (the start of electronic collection of records), to Dec 31, 2017, were provided by the Western Cape Provincial Health Data Centre.^12^ This service links patient information from several routine health information systems, including hospital discharge summaries (with International Classification of Diseases, 10th Revision [ICD-10] diagnoses) and pharmacy records, via a unique identifier. All ART programmes participating in IeDEA have ethics approval to examine long-term outcomes of patients at their facilities through linkage to other datasets, and to contribute de-identified data to the IeDEA Data Centre. The Western Cape Department of Health approved the linkage of ART and mental health records. The Human Research Ethics Committee of the University of Cape Town, South Africa, and the Cantonal Ethics Committee of the Canton of Bern, Switzerland, granted permission for analysis of the linked database.

### Treatment programmes and patients

Gugulethu Community Health Clinic (CHC), Tygerberg Academic Hospital, and the Khayelitsha ART programme, all situated in Cape Town, provide public HIV treatment according to national guidelines.^13^ Gugulethu CHC and the Khayelitsha ART programme provide primary care to the townships of Gugulethu and Khayelitsha, while Tygerberg Academic Hospital is a tertiary care, referral hospital that generally manages patients with severe illness. In the Western Cape province, primary care facilities are the first point of care for individuals with both common mental disorders and stable, severe mental disorders. Individuals with common and severe mental disorders requiring either admission or specialised services are referred to secondary and tertiary care facilities. For our study, people living with HIV aged 15 years or older at ART initiation, who had initiated ART at Gugulethu CHC, Tygerberg Academic Hospital, or one of the health centres of the Khayelitsha ART programme (Khayelitsha [Site B] CHC, Michael Mapongwana CHC, and Nolungile CHC) were eligible for analysis.

### Procedures

We followed up patients from ART initiation or Jan 1, 2010, whichever occurred later, to transfer, death, IeDEA database closure (Dec 31, 2017), or the end of their tenth year on ART. Mortality was documented by the ART programmes, which we updated by linking ART records to mortality surveillance data from the South African National Population Register^11,12^ up to Dec 31, 2017. The National Population Register classified the underlying cause of death as unnatural (ICD-10 codes V01-Y99) or natural (death due to natural disease per the ICD-10). We defined HIV treatment outcomes as retention in care with viral load suppression (VLS; viral load <1000 copies per mL), retention in care with non-suppressed viral load (NVL; viral load ≥1000 copies per mL), and loss to follow-up (LTFU; >180 days late for a clinic visit at closure of the IeDEA database. Patients who were not LTFU, deceased, or transferred to a different ART programme (censored on transfer) were classified as retained in care. Viral rebound was defined as transitioning from VLS to NVL. Patients without a viral load measurement at ART initiation were initially classified as NVL. HIV treatment outcomes were updated whenever the treatment status of a patient changed. We assessed the WHO clinical stage and CD4 cell count of patients at initiation of ART. Patient age was assessed at the beginning of each year. On each programme, HIV RNA viral load was measured 4–6 months after ART initiation, 12 months after ART initiation, and annually thereafter, as per national and provincial guidelines.^13^ The year of ART initiation was categorised as 2004–07, 2008–11, 2012–14, and 2015–17, with years grouped according to ART guideline periods.

Our primary exposure was history of mental illness. We classified patients as having a history of mental illness if they had ever received psychiatric medication (antipsychotics [WHO Anatomical Therapeutic Chemical code N05A], anxiolytics [N05B], hypnotics and sedatives [N05C], antidepressants [N06A], or psychostimulants [N06B]) or had ever been admitted to hospital for a mental disorder (ICD-10 codes F00-F99) or to a psychiatric ward or facility. Reason for hospitalisation was classified according to the final diagnosis recorded in hospital discharge summaries. Reasons for hospitalisation were substance use disorder (ICD-10 diagnoses F10-F19), psychotic disorder (F20-F29), affective disorder (F30-F39), and anxiety and related disorders (F40-F48). The remaining ICD code categories were grouped together as unspecified (F99) or as other (F00-F09, F49-F98). We classified pharmacy records according to the Anatomical Therapeutic Chemical classification system, as antipsychotic (code N05A), anxiolytic (N05B), antidepressant (N06A), or other psychiatric medication (N05 or N06).

### Statistical analysis

We calculated hazard ratios (HRs) and cause-specific HRs with 95% CIs for factors associated with excess mortality from all causes, natural causes, and unnatural causes in people with a history of mental illness using univariable Cox proportional hazard models. History of mental illness was defined as a time-varying binary variable. Patients were considered unaffected by mental illness (unexposed) until they had received their first mental health treatment; thereafter, patients were considered to be exposed. We subsequently examined excess mortality in people with a history of mental illness in three adjusted multivariable Cox proportional hazard models. Model 1 adjusted for age as a time-varying covariate (15–24 years, 25–34 years, 35–44 years, 45–54 years, 55–64 years, and ≥65 years), sex, treatment programme, and year of ART initiation. Model 2 adjusted for all variables included in model 1, and CD4 cell count (missing, <100 cells per μL, 100–199 cells per μL, 200–349 cells per μL, 350–499 cells per μL, and ≥500 cells per μL according to WHO thresholds) and WHO clinical stage (stage 1, 2, 3, 4, or missing) at ART initiation. Model 3 adjusted for all variables included in model 2, and HIV treatment outcome as a time-varying covariate (retained in care with VLS, retained in care with NVL, and LTFU). We did a post-hoc sensitivity analysis using alternative definitions of mental illness. In this analysis, we considered patients to be affected by mental illness (exposed) for 1, 2, 3, 4, and 5 years after mental health treatment (ie, dispensing of psychiatric medication or hospital admission for mental disorder), and as unexposed thereafter. Patients who never received mental health treatment were considered unexposed. We calculated aHRs and 95% CIs for associations between each of the five exposure variables and all-cause mortality using multi variable Cox proportional hazards models adjusted for model 2 covariates.

Additionally, we examined excess all-cause mortality in people with a history of mental illness according to HIV treatment outcomes using a multistate model.^14,15^ The model allowed for transitions between three states representing HIV treatment outcomes (retained in care with NVL [state 1], retained in care with VLS [state 2], and LTFU [state 3]). From each of these states, patients could transition to death (state 4). We used Cox proportional hazard models with stratified non-parametric baseline hazards to estimate transition probabilities and cause-specific aHRs and 95% CIs for associations between exposure variables and transition rates for each transition in the multistate model.^15^ Multistate models were adjusted for age, sex, WHO clinical stage and CD4 cell count at ART initiation, year of ART initiation, cumulative time with NVL before entering a state, and treatment programme. We plotted transition probabilities (expressed as percentage) describing the chances of patients with and without a history of mental illness of being in each of the four states of the multistate model during 10 years on ART. We used this multistate model to do a prespecified subgroup analysis, in patients who had received antipsychotics, antidepressants, or anxiolytics; and in patients who had received an ICD-10 diagnosis for substance use disorder, psychotic disorder, affective disorder, or anxiety disorder. Post-hoc subgroup analyses were done in patients who had been hospitalised for any mental disorder; in patients who had never been hospitalised for a mental disorder but had received psychiatric medication; in patients who received mental health treatment (ie, psychiatric medication or hospitalisation for a mental disorder) during ART; and in patients who received mental health treatment before but not during ART. In the analysis of patients who had received antipsychotics, antidepressants, or anxiolytics, patients without a history of use of psychiatric medication were the reference group. The reference group in all other subgroup analyses was people without a history of mental illness. We assessed the proportional hazards assumption using Schoenfeld residuals and visual inspection of log-log plots. All Cox models were adjusted for treatment programme by stratificaton of the baseline hazard function because this covariate did not satisfy the proportional hazards assumption. We included dummy variables to handle missing covariate data. Data were managed and analysed with Stata (version 15), R software, and the R package mstate.^15^

### Role of the funding source

The funder of the study had no role in study design, data collection, data analysis, data interpretation, or writing of the report. The corresponding author had full access to all the data in the study and had final responsibility for the decision to submit for publication.

## Results

10 587 patients from Gugulethu, 44 240 from Khayelitsha, and 3837 from Tygerberg were followed up for a median duration of 4·3 years (IQR 2·1–6·4). Of 58 664 patients, 2927 (5·0%) had a history of mental illness. 41 453 (70·7%) patients were women. The median age at the start of follow-up was 33 years (IQR 28–40). 4010 patients (6·8%) died during follow-up, of whom 3139 (78·3%) died from a natural cause, 216 (5·4%) from an unnatural cause, and 655 (16·3%) from an unknown cause (table 1). Of 2927 patients with a history of mental illness, 2565 (87·6%) had received psychiatric medication (of whom, 1561 [60·9%] had received antipsychotics) and 923 (31·5%) had been hospitalised for a mental disorder (table 2).

**Figure 1:**
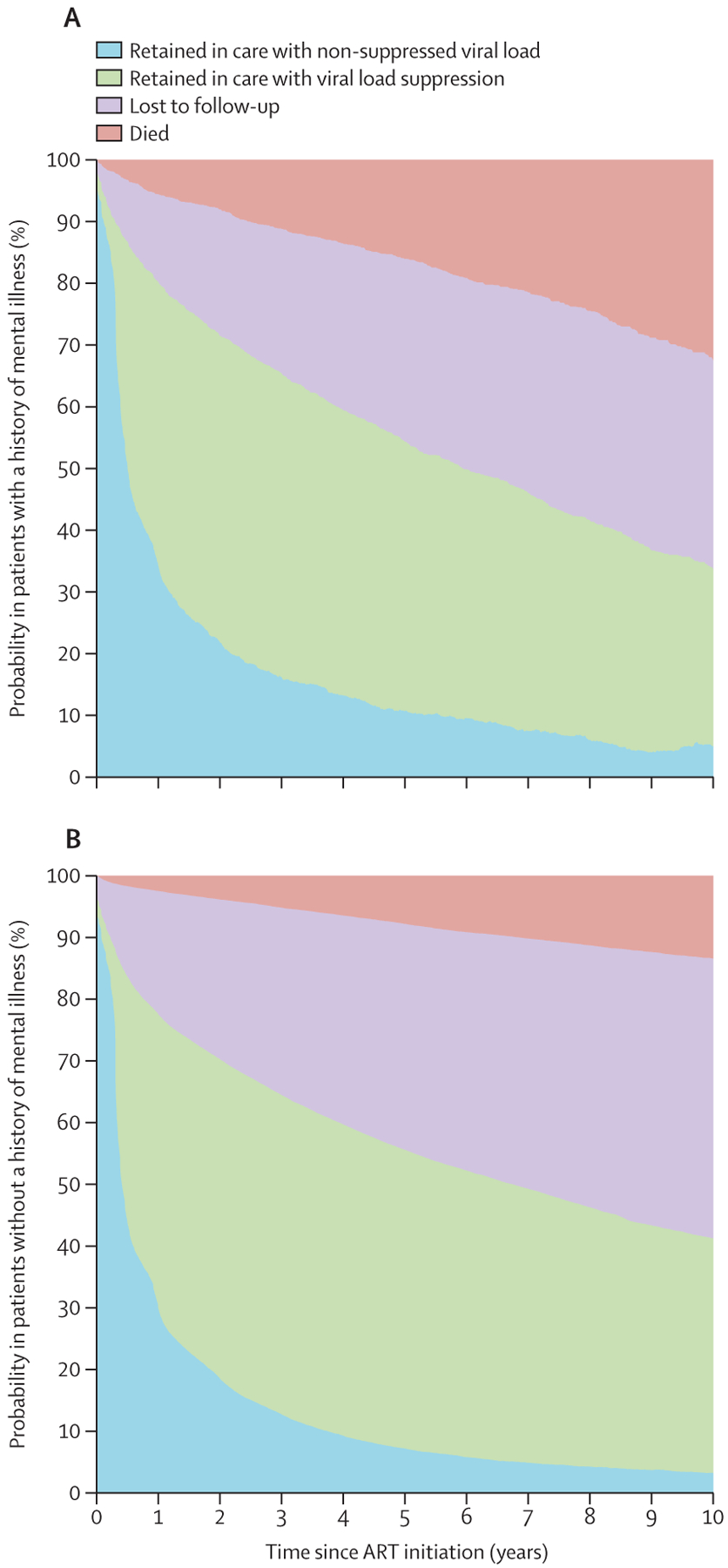
Probability of HIV treatment outcomes and all-cause mortality during 10 years of ART for patients with a history of mental illness (A) and without a history of mental illness (B) Percentages represent the probability of being in each of the four states of the multistate model during 10 years on ART. ART=antiretroviral therapy.

Figure 1 compares the probability of HIV treatment outcomes and all-cause mortality within our multistate model in patients with and without a history of mental illness over 10 years on ART. Among patients with a history of mental illness, at 10 years after ART initiation, the probability of death was 32·0% (95% CI 28·9–35·1). The probabilities of LTFU, retention in care with VLS, and retention in care with NVL were 34·0% (30·8–37·1), 28·9% (26·2–31·7), and 5·1% (3·5–6·7), respectively (figure 1A, appendix p 1). Among patients with no history of mental illness, at 10 years after ART initiation, the probabilities of death, LTFU, retention with VLS, and retention with NVL were 13·4% (12·9–13·9), 45·3% (44·6–46·0), 38·0% (37·4–38·7), and 3·2% (2·9–3·5), respectively (figure 1B, appendix p 1). Transition probabilities of ART outcomes over 10 years for patients with and without a history of mental illness for each treatment programme are shown in the appendix (pp 2, 5). Overall, mortality was higher in the Tygerberg tertiary care hospital than in the two primary care programmes. In all treatment programmes, the probability of death 10 years after ART initiation was higher in people with a history of mental illness than in those without.

In our univariate analysis, history of mental illness was associated with all-cause mortality (HR 3·05 [95% CI 2·75–3·37]), mortality from natural causes (3·14 [2·81–3·51]), and mortality from unnatural causes (2·15 [1·29–3·56]; table 3, appendix p 3). Rates of excess all-cause mortality in patients with a history of mental illness and in those who initiated ART at WHO clinical stage 4 were similar (appendix p 3). In a multivariable analysis adjusted for age, sex, year of ART initiation, and treatment programme (model 1), history of mental illness was associated with all-cause mortality (aHR 2·98 [95% CI 2·69–3·30]), mortality from natural causes (3·00 [2·69–3·36]), and mortality from un natural causes (2·10 [1·27–3·49]; table 3, appendix p 3). The associations between history of mental illness and mortality were marginally attenuated in a multivariable model adjusted for age, sex, year of ART initiation, treatment programme, and CD4 cell count and WHO clinical stage at ART initiation (model 2). With additional adjustment for retention in HIV care with or without VLS and LTFU (model 3), history of mental illness remained associated with all-cause mortality (aHR 2·73 [95% CI 2·46–3·02]) and mortality from natural causes (2·75 [2·46–3·08]; table 3, appendix p 3). The association between mental illness and all-cause mortality (adjusted for age, sex, year of ART initiation, treatment programme, and CD4 cell count and WHO clinical stage at ART initiation) was greater (aHR 3·49 [95% CI 3·09–3·94]) in sensitivity analysis, when we considered patients to be affected by mental illness (exposed) for 1 year after each mental health treatment (ie, dispensing of psychiatric medication or hospital admission for mental disorder) and as unexposed thereafter. Associations were gradually attenuated when the 1-year period was extended to 2, 3, 4, and 5 years (appendix p 4).

**Figure 2:**
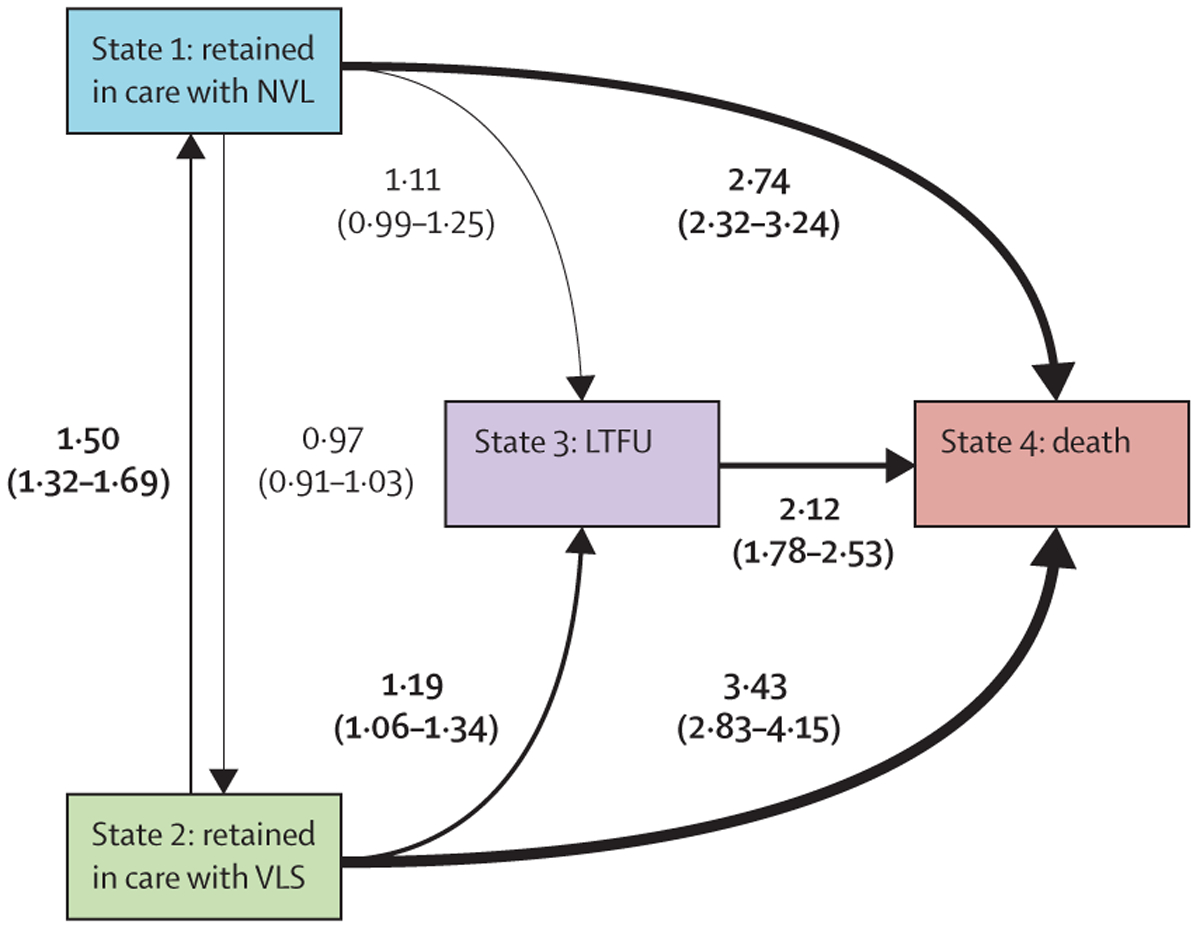
aHRs comparing mortality and HIV treatment outcomes between patients with and without a history of mental illness aHRs (395% CIs) are for the transition rates of patients with a history of mental illness versus those without for each transition of the multistate model. Patients without a history of mental illness were the reference group. Arrow width is proportional to the strength of the association. HRs were adjusted for age, sex, year of ART initiation, cumulative time with NVL, CD4 cell count and WHO clinical stage at ART initiation, and treatment programme. Values with 95% CIs that do not include 1 are shown in bold. aHR=adjusted hazard ratio. ART=antiretroviral therapy. LTFU=loss to follow-up. NVL=non-suppressed viral load. VLS=viral load suppression.

Figure 2 shows aHRs and 95% CIs for the transition rates of patients with a history of mental illness versus those without for each transition of the multistate model. Excess all-cause mortality in people with a history of mental illness occurred independently of HIV treatment outcomes. Associations between history of mental illness and all-cause mortality were greatest in patients retained in care with VLS (aHR 3·43 [95% CI 2·83–4·15]), followed by patients retained in care with NVL (2·74 [2·32–3·24]), and smallest in those LTFU (2·12 [1·78–2·53]). History of mental illness was also associated with increased rates of viral rebound (VLS to NVL, 1·50 [1·32–1·69]) and increased rates of LTFU in people with VLS (1·19 [1·06–1·34]).

In subgroup analyses, associations between mortality, adverse HIV treatment outcomes (viral rebound and LTFU), and mental health treatment were stronger in patients who received mental health treatment during ART (appendix p 6) than in those who received mental health treatment before but not during ART (appendix p 7). Patients who received mental health treatment before but not during ART were at a higher risk of mortality but not a higher risk of LTFU or viral rebound than patients without a history of mental illness (appendix p 7). Patients who had been admitted to hospital for a mental disorder (appendix p 8) and patients who had never been admitted for a mental disorder but had received psychiatric medication (appendix p 9) had similar rates of excess all-cause mortality, viral rebound, and LTFU when compared with those without a history of mental illness. Among patients who received psychiatric medications, those who received antipsychotics had higher rates of excess all-cause mortality, viral rebound, and LTFU than those who received antidepressants or anxiolytics, when patients without a history of medication were the reference group (appendix p 10). Among patients who received psychiatric diagnoses, those diagnosed with substance use disorders had the greatest risk increase of unfavourable virological outcomes when compared with patients without a history of mental illness (appendix p 11). Notably, patients with substance use disorders had the lowest rate of achieving VLS and the highest rate of viral rebound. Increased rates of unfavourable virological outcomes were also seen in patients with affective or anxiety disorders, and rates of all-cause mortality were increased in patients with psychotic or anxiety disorders, compared with patients without a history of mental illness (appendix p 11).

## Discussion

To our knowledge, this study is the first to quantify mortality associated with mental illness in HIV-positive people in South Africa, where the HIV-positive population accounts for 20% of all cases globally (2018 UNAIDS estimate). This study showed substantial excess mortality and an increased rate of adverse HIV treatment outcomes in people with a history of mental illness versus those without. People with a history of mental illness had marginally higher rates of LTFU and viral rebound, around 3-times higher rates of mortality from all causes, and natural causes, and around 2-times higher rates of mortality from unnatural causes, than individuals with no history of mental illness. Excess mortality in people with mental illness was independent of retention in HIV care, VLS, and the stage of HIV disease progression at ART initiation. Excess death rates in patients with a history of mental illness and in those who initiated ART at WHO clinical stage 4 were similar.

The pattern of excess mortality observed in our study is consistent with previous studies of excess mortality associated with mental illness in HIV-positive populations. Two studies in the USA showed that risk of mortality was 2–4 times higher in HIV-positive women with depression than in those without.^7,8^ A study in Tanzania found that in HIV-positive women, mortality risk was 2 times higher in women with symptoms of depression than in those without.^10^ A nationwide population-based cohort study in Denmark showed that mortality in people with schizophrenia and HIV was 3 times higher than mortality among people with HIV alone.^9^

Strong evidence shows that people with mental illness are less adherent to theraueptic regimens such as ART than people without mental illness.^6^ We expected that poor retention and adherence to ART would be an important driver of excess mortality in people living with HIV and mental illness. However, our study provides little support for this particular hypothesis. Excess mortality in people with mental illness occurred independently of HIV treatment outcomes. The largest mortality gap between patients with and without a history of mental illness was seen in patients retained on ART with VLS. As such, we believe that other underlying mechanisms are responsible for the excess mortality in people with mental illness.

One mechanism that contributes to excess mortality in people with mental disorders is excess mortality from unnatural causes. Although our study confirmed previous reports, which showed people with mental disorders to have an increased risk of unnatural death,^16,17^ we found that excess mortality from unnatural causes only accounted for a small proportion of the mortality gap in people with mental disorders. Consistent with previous studies, we found that most premature deaths in people with mental disorders were due to natural causes.^2^

Increased rates of physical illness and inadequate health care contribute to substantially higher than average rates of death from natural causes in people with mental disorders.^18^ Strong evidence suggests that some mental illnesses, such as depression and anxiety, are independent risk factors of physical illnesses including cardiovascular and metabolic diseases.^19–22^ Further reasons that might lead to increased incidence of physical illness in people with mental illness include side-effects of psychiatric medication (eg, weight gain and increased diabetogenic and cerebrovascular disease risk^22^), and high rates of adverse lifestyle behaviours (eg, poor diet, low physical activity, smoking, alcohol use, or drug use).^18^ Furthermore, disparities in health care might contribute to excess mortality in people with mental illness.^18,23^ Data from developed countries show that people with mental illness are less likely to receive physical examinations, screening, diagnostic tests, or the recommended standard of care for metabolic and cardiovascular diseases.^18,22,23^ Possible mechanisms underlying these disparities include differences in health-care seeking behaviour, structural barriers limiting access to health care for people with mental illness, misattribution of physical conditions as psychosomatic symptoms, implicit physician bias, and stigmatising attitudes of health-care providers towards people with mental illness.^23,24^

Although findings support a causal relationship between mental disorders and mortality, caution in interpreting these associations is warranted. Associations between mental disorders and mortality could also have a non-causal origin and be the result of confounding. Physical comorbidity and socioeconomic status are important potential confounding factors. We adjusted for WHO clinical stage and CD4 cell count at ART initiation. These variables are generally accepted as good proxies for the most relevant physical ilnesses in people living with HIV (eg, pulmonary and extra pulmonary tuberculosis, and AIDS-defining cancers). However, we were limited by the data available for inclusion in our analyses and could not adjust for other potentially relevant comorbidities. Furthermore, we did not adjust for incident comorbidities after ART initiation because physical comorbidity is on the causal path from exposure (mental illness) to outcome (mortality).^18–22^ Adjustment for such intermediate variables would lead to overadjustment bias, resulting in substantial underestimation of excess mortality in mental illness.^25^ Furthermore, we had no data on the socioeconomic status of patients and could not adjust for this potential confounder. However, we believe that in our study, socioeconomic status was not an important confounder because our study population had no major socioeconomic differences that could explain the large mortality gap in people with mental disorders. Most people with and without mental disorders were enrolled in the public-sector ART programmes at Gugulethu and Khayelitsha. These townships are among the poorest areas in Cape Town and people with higher socioeconomic status are unlikely to access public-sector HIV care in these settings.

Although evidence for the effectiveness of strategies to improve survival in people with mental illness is scarce,^18^ we believe that interventions addressing the multiple underlying drivers of excess mortality hold promise for closing the mortality gap between people with and without mental illness. Optimal dosing and screening for side-effects of psychiatric medication, screening and management of common chronic physical comorbidities, and interventions addressing adherence to therapeutic regimens, health equity, lifestyle behaviours, and stigma against people with mental illness could reduce the burden of physical illness among people with mental illness.^18,23,26,27^ Interventions should also include suicide prevention and ideally address social determinants of health.^18,24^ Our study also suggests that individuals who received antipsychotic medication are a highly vulnerable population requiring specific clinical attention.

The large sample size, long follow-up, multicohort design, inclusion of primary and tertiary care ART programmes, and data from multiple sources are important strengths of this study that add to the robustness and generalisability of our findings. The ascertainment of mortality by linking ART records to the South African National Population Register was a further strength that enabled us to assess excess mortality in people with mental illness among people who were not retained in HIV care.

Our results should be considered in view of several limitations. We classified the mental health status of patients on the basis of routinely collected records of mental health treatments. We used hospital admissions for mental health disorders and receipt of prescriptions for psychiatric medications as the best available proxies for mental illness. As such, patients receiving non-pharmacological outpatient interventions such as psychotherapy might have been overlooked, and patients who received psychiatric medications for indications not related to mental health might have been misclassified. Globally, most people living with mental illness do not have access to mental health treatment.^28^ In our study, people who were affected by mental disorders but remained untreated would have been misclassified as having no history of mental illness. Misclassification of untreated people with mental illness would have led to bias towards the null hypothesis. Therefore, our study might underestimate associations between mental illness, mortality, and adverse HIV treatment outcomes. People with mild forms of mental disorders might be less likely to be diagnosed and treated, and thus people with severe mental illness might be over-represented in our sample. Our study might therefore overestimate associations between mental illness, mortality, and adverse HIV treatment outcomes. ICD-10 diagnoses were only available from hospital discharge summaries and only for a small percentage of the individuals with mental illness. Our subgroup analysis of associations between particular types of disorders and HIV treatment outcomes has to be interpreted with caution as only the more severe cases that required hospitalisation were likely to be included in this analysis. Throughout our analyses, we could not adjust HIV programme data for undocumented transfers and we did not consider intermittent treatment interruptions in patients who returned to care as LTFU periods.^29^

Although our results need to be interpreted in the context of some limitations, our study is one of the few to assess associations between mental illness, mortality, and other HIV-related outcomes. Mental illness was independently associated with decreased retention on ART, decreased VLS, and increased mortality from natural and unnatural causes. These findings suggest that excess mortality in people with mental illness cannot be solely attributed to the negative effect of mental illness on HIV treatment. Interventions to reduce excess mortality in people with mental illness should go beyond support for treatment adherence and address the complex physical and mental health-care needs of people living with HIV and mental illness.

## Supplementary Material

1

## Figures and Tables

**Table 1:** Patient characteristics by history of mental illness

	No history of mental illness (n=55 737)	History of mental illness (n=2927)	Total (n=58 664)
**Characteristics at ART initiation**			
Sex			
Male	16319(29.3%)	892 (30.5%)	17211(29.3%)
Female	39418(70.7%)	2035 (69.5%)	41453 (70.7%)
CD4 count, ceils per μL			
<100	10579(19.0%)	643 (22.0%)	11222(19.1%)
100–199	12 793(23.0%)	678 (23.2%)	13471(23.0%)
200–349	12 884(23.1%)	611 (20.9%)	13495(23.0%)
350–499	4289 (7.7%)	199 (6.8%)	4488 (7.7%)
≥500	2538 (4.6%)	123 (4.2%)	2661 (4.5%)
Missing	12 654 (22.7%)	673 (23.0%)	13327(22.7%)
Median (IQR)	186 (101–293)	171(87–281)	186 (101–292)
WHO clinical stage			
1	18794(33.7%)	666 (22.8%)	19460(33.2%)
2	10163(18.2%)	483 (16.5%)	10 646 (18.1%)
3	17296(31.0%)	959 (32.8%)	18255(31.1%)
4	7445 (13.4%)	664 (22.7%)	8109 (13.8%)
Missing	2039 (3.7%)	155 (5.3%)	2194 (3.7%)
Year of ART initiation			
2004–07	9112 (16.3%)	443 (15.1%)	9555 (16.3%)
2008–11	17250 (30.9%)	1027 (35.1%)	18277(31.2%)
2012–14	16942(30.4%)	919 (31.4%)	17861(30.4%)
2015–17	12 433(22.3%)	538 (18.4%)	12 971(22.1%)
**Age at start of follow-up, years**			
15–24	6129 (11.0%)	233 (8.0%)	6362 (10.8%)
25–34	25196(45.2%)	1179 (40.3%)	26375(45.0%)
35–44	16496(29.6%)	910 (31.1%)	17406(29.7%)
45–54	6150 (11.0%)	444 (15.2%)	6594 (11.2%)
≥55	1766 (3.2%)	161 (5.5%)	1927 (3.3%)
Median (IQR)	33 (28–40)	35 (29–42)	33 (28–40)
**HIV treatment outcome at end of follow-up**			
Retained in care	26465(47.5%)	1264(43.2%)	27729 (47.3%)
LTFU	17946(32.2%)	792 (27.1%)	18738(31.9%)
Transferred	7769 (13.9%)	418 (14.3%)	8187 (14.0%)
Died	3557(6.4%)	453 (15.5%)	4010 (6.8%)
Natural cause*	2761 (77.6%)	378 (83.4%)	3139 (78.3%)
Unnatural cause*	199 (5.6%)	17 (3.8%)	216 (5.4%)
Unknown cause*	597 (16.8%)	58 (12.8%)	655 (16.3%)
HIV treatment outcome before death*			
Retained in care with non-suppressed viral load	1352 (38.0%)	171 (37.7%)	1523 (38.0%)
Retained in care with viral load suppression	960 (27.0%)	131 (28.9%)	1091 (27.2%)
LTFU	1245 (35.0%)	151 (33.3%)	1396 (34.8%)

Data are n (%) unless otherwise stated. Percentages do not always add up to 100% due to rounding. Patients with documented pharmacological mental health treatment or mental health-related hospital admission were classified as having a history of mental illness. ART=antiretroviral therapy. LTFU=loss to follow-up.

* Denominators for percentages are total deaths in that group.

**Table 2:** Mental health treatment received by patients with a history of mental illness

	Number of patients (%)
Any mental health treatment	2927 (100.0%)
Psychiatric medication	2565 (87.6%)
Antipsychotics (N05A)	1561 (60.9%)*
Anxiolytics (N05B)	815 (31.8%)*
Antidepressants (N06A)	1159 (45.2%)*
Other psychiatric medication (N05 or N06)	13 (0.5%)*^†^
Mental health-related hospital admission	923 (31.5%)
Hospitalised for substance use disorder (F10–F19)	103 (11.2%)^‡^
Hospitalised for psychotic disorder (F20–F29)	182 (19.7%)^‡^
Hospitalised for affective disorder (F30–F39)	150 (16.3%)^‡^
Hospitalised for anxiety and related disorders (F40–F48)	40 (4.3%)^‡^
Hospitalised for an unspecified (F99) or other mental disorder (F00–F09, F49–F98)	276 (29.9%)^‡^
Hospitalised to psychiatric ward for unknown disorder	356 (38.6%)^‡^

Patients with documented pharmacological mental health treatment or mental health-related hospital admission were classified as having a history of mental illness. Categories in the table are not mutually exclusive. Psychiatric medications were classified according to the Anatomical Therapeutic Chemical classification system and mental and substance use disorders according to the International Classification of Diseases, 10th Revision.

* Percentages are out of those who received psychiatric medication.

† Nine patients received hypnotics and sedatives (N05C) and four received psychostimulants (N06B).

‡ Percentages are out of those admitted to hospital.

**Table 3:** HRs and cause-specific HRs for excess mortality in patients with a history of mental illness

	All-cause mortality,* HR (95% Cl)	Mortality from natural cause,^†^ HR(95%CI)	Mortality from unnatural cause,^†^HR(95%CI)
Univariable analyses	3.05 (2.75–3.37)	3.14(2.81–3.51)	2.15 (1.29–3.56)
Multivariable analyses			
Model 1	2.98 (2.69–3.30)	3.00 (2.69–3.36)	2.10 (1.27–3.49)
Model 2	2.76 (2.50–3.06)	2.78 (2.48–3.11)	2.05 (1.23–3.41)
Model 3	2.73 (2.46–3.02)	2.75 (2.46–3.08)	2.07 (1.24–3.44)

Model 1 adjusted for age, sex, year of ART initiation, and treatment programme; model 2 adjusted for all variables in model 1, and WHO clinical stage and CD4 cell count at ART initiation; model 3 adjusted for all variables in model 2 and HIV treatment status (retained with viral load suppression, retained with non-suppressed viral load, or loss to follow-up). History of mental illness, age, and HIV treatment status were modeled as time-varying covariates. HR=hazard ratio. ART=antiretroviral therapy.

* HRs for excess mortality in patients with a history of mental illness compared with patients without a history of mental illness.

† Cause-specific HRs for excess mortality in patients with a history of mental illness compared with patients without a history of mental illness.

## Research in context

### Evidence before this study

We searched PubMed for reports published in any language between Jan 1, 2000, and Aug 1, 2019, with the search terms (“excess mortality” OR “premature mortality”) AND (“mental illness” OR “mental disorder” OR “depression” OR “anxiety” OR “bipolar” OR “psychotic” OR “schizophrenia” OR “substance use” OR “alcohol use” OR “trauma” OR “post-traumatic stress”) in the title or abstract. Excess mortality in people with mental disorders is well documented for high-income countries, but data are scarce for low-income and middle-income countries and for HIV-positive populations. Additionally, the Global Burden of Disease Study does not capture mortality attributable to mental disorders in its disability-adjusted life-year or years of life lost metrics.

### Added value of this study

Our study is one of a few to quantify mortality associated with mental illness in HIV-positive populations of low-income and middle-income countries and, to our knowledge, the first to do so in South Africa, where the HIV-positive population accounts for 20% of all cases globally (2018 UNAIDS estimate).

We analysed a large observational dataset after integrating longitudinal data on antiretroviral therapy (ART), mental health treatment, and mortality surveillance data on almost 60 000 people receiving HIV care in public-sector ART programmes in Cape Town. The risk of all-cause mortality was around 3-times higher in people with a history of mental illness than in individuals without. Although history of mental illness was associated with decreased retention on ART and decreased viral load suppression, our study showed that excess mortality in people with mental illness occurred independently of HIV treatment success.

### Implications of all the available evidence

Our study shows that excess mortality in people with mental disorders is an urgent public health problem in settings with a high prevalence of HIV. Substantial investments in the development, evaluation, and implementation of interventions are needed to close the mortality gap for people with mental illness. Interventions should go beyond supporting adherence to ART to address the complex physical and mental health-care needs of people living with HIV and mental illness.

## References

[1] Herman AA, Stein DJ, Seedat S, Heeringa SG, Moomal H, Williams DR. The South African Stress and Health (SASH) study: 12-month and lifetime prevalence of common mental disorders. S Afr Med J 2009; 99: 339–44. 19588796 PMC3191537

[2] Walker ER, McGee RE, Druss BG. Mortality in mental disorders and global disease burden implications: a systematic review and meta-analysis. JAMA Psychiatry 2015; 72: 334–41. 25671328 10.1001/jamapsychiatry.2014.2502 PMC4461039

[3] Mogga S, Prince M, Alem A, Outcome of major depression in Ethiopia: population-based study. Br J Psychiatry 2006; 189: 241–46. 16946359 10.1192/bjp.bp.105.013417

[4] Teferra S, Shibre T, Fekadu A, Five-year mortality in a cohort of people with schizophrenia in Ethiopia. BMC Psychiatry 2011; 11: 165. 21985179 10.1186/1471-244X-11-165 PMC3207944

[5] Myer L, Smit J, Roux LL, Parker S, Stein DJ, Seedat S. Common mental disorders among HIV-infected individuals in South Africa: prevalence, predictors, and validation of brief psychiatric rating scales. AIDS Patient Care STDS 2008; 22: 147–58. 18260806 10.1089/apc.2007.0102

[6] Uthman OA, Magidson JF, Safren SA, Nachega JB. Depression and adherence to antiretroviral therapy in low-, middle- and high-income countries: a systematic review and meta-analysis. Curr HIV/AIDS Rep 2014; 11: 291–307. 25038748 10.1007/s11904-014-0220-1 PMC4359613

[7] Ickovics JR, Hamburger ME, Vlahov D, Mortality, CD4 cell count decline, and depressive symptoms among HIV-seropositive women: longitudinal analysis from the HIV Epidemiology Research Study. JAMA 2001; 285: 1466–74. 11255423 10.1001/jama.285.11.1466

[8] Cook JA, Grey D, Burke J, Depressive symptoms and AIDS-related mortality among a multisite cohort of HIV-positive women. Am J Public Health 2004; 94: 1133–40. 15226133 10.2105/ajph.94.7.1133 PMC1448411

[9] Helleberg M, Pedersen MG, Pedersen CB, Mortensen PB, Obel N. Associations between HIV and schizophrenia and their effect on HIV treatment outcomes: a nationwide population-based cohort study in Denmark. Lancet HIV 2015; 2: e344–50. 26423377 10.1016/S2352-3018(15)00089-2

[10] Sudfeld CR, Kaaya S, Gunaratna NS, Depression at antiretroviral therapy initiation and clinical outcomes among a cohort of Tanzanian women living with HIV. AIDS 2017; 31: 263–71. 27835614 10.1097/QAD.0000000000001323 PMC5177498

[11] Chammartin F, Dao Ostinelli CH, Anastos K, International epidemiology databases to evaluate AIDS (IeDEA) in sub-Saharan Africa, 2012–2019. BMJ Open 2020; 10: e035246. 10.1136/bmjopen-2019-035246 PMC7232622 32414825

[12] Boulle A, Heekes A, Tiffin N, Data centre profile: the Provincial Health Data Centre of the Western Cape province, South Africa. Int J Popul Data Sci 2019; 4: 6. 10.23889/ijpds.v4i2.1143 PMC7482518 32935043

[13] Meintjes G, Moorhouse MA, Carmona S, Adult antiretroviral therapy guidelines 2017. South Afr J HIV Med 2017; 18: 776. 29568644 10.4102/sajhivmed.v18i1.776 PMC5843236

[14] Crowther MJ, Lambert PC. Parametric multistate survival models: flexible modelling allowing transition-specific distributions with application to estimating clinically useful measures of effect differences. Stat Med 2017; 36: 4719–42. 28872690 10.1002/sim.7448

[15] de Wreede LC, Fiocco M, Putter H. The mstate package for estimation and prediction in non- and semi-parametric multi-state and competing risks models. Comput Methods Programs Biomed 2010; 99: 261–74. 20227129 10.1016/j.cmpb.2010.01.001

[16] Too LS, Spittal MJ, Bugeja L, Reifels L, Butterworth P, Pirkis J. The association between mental disorders and suicide: a systematic review and meta-analysis of record linkage studies. J Affect Disord 2019; 259: 302–13. 31450139 10.1016/j.jad.2019.08.054

[17] Crump C, Sundquist K, Winkleby MA, Sundquist J. Mental disorders and risk of accidental death. Br J Psychiatry 2013; 203: 297–302. 23969485 10.1192/bjp.bp.112.123992

[18] Liu NH, Daumit GL, Dua T, Excess mortality in persons with severe mental disorders: a multilevel intervention framework and priorities for clinical practice, policy and research agendas. World Psychiatry 2017; 16: 30–40. 28127922 10.1002/wps.20384 PMC5269481

[19] Batelaan NM, Seldenrijk A, Bot M, Balkom AJLM, Penninx BWJH. Anxiety and new onset of cardiovascular disease: critical review and meta-analysis. Br J Psychiatry 2016; 208: 223–31. 26932485 10.1192/bjp.bp.114.156554

[20] Lichtman JH, Bigger JT Jr, Blumenthal JA, Depression and coronary heart disease: recommendations for screening, referral, and treatment: a science advisory from the American Heart Association Prevention Committee of the Council on Cardiovascular Nursing, Council on Clinical Cardiology, Council on Clinical Cardiology, Council on Epidemiology and Prevention, and Interdisciplinary Council on Quality of Care and Outcomes Research: endorsed by the American Psychiatric Association. Circulation 2008; 118: 1768–75. 18824640 10.1161/CIRCULATIONAHA.108.190769

[21] Rotella F, Mannucci E. Depression as a risk factor for diabetes: a meta-analysis of longitudinal studies. J Clin Psychiatry 2013; 74: 31–37. 23419223 10.4088/JCP.12r07922

[22] De Hert M, Correll CU, Bobes J, Physical illness in patients with severe mental disorders. I. Prevalence, impact of medications and disparities in health care. World Psychiatry 2011; 10: 52–77. 21379357 10.1002/j.2051-5545.2011.tb00014.x PMC3048500

[23] De Hert M, Cohen D, Bobes J, Physical illness in patients with severe mental disorders. II. Barriers to care, monitoring and treatment guidelines, plus recommendations at the system and individual level. World Psychiatry 2011; 10: 138–51. 21633691 10.1002/j.2051-5545.2011.tb00036.x PMC3104888

[24] Thornicroft G, Rose D, Kassam A. Discrimination in health care against people with mental illness. Int Rev Psychiatry 2007; 19: 113–22. 17464789 10.1080/09540260701278937

[25] Schisterman EF, Cole SR, Platt RW. Overadjustment bias and unnecessary adjustment in epidemiologic studies. Epidemiology 2009; 20: 488–95. 19525685 10.1097/EDE.0b013e3181a819a1 PMC2744485

[26] Barber S, Thornicroft G. Reducing the mortality gap in people with severe mental disorders: the role of lifestyle psychosocial interventions. Front Psychiatry 2018; 9: 463. 30323773 10.3389/fpsyt.2018.00463 PMC6172296

[27] Kohrt BA, Jordans MJD, Turner EL, Reducing stigma among healthcare providers to improve mental health services (RESHAPE): protocol for a pilot cluster randomized controlled trial of a stigma reduction intervention for training primary healthcare workers in Nepal. Pilot Feasibility Stud 2018; 4: 36. 29403650 10.1186/s40814-018-0234-3 PMC5781273

[28] Patel V, Saxena S, Lund C, The *Lancet* Commission on global mental health and sustainable development. Lancet 2018; 392: 1553–98. 30314863 10.1016/S0140-6736(18)31612-X

[29] Haas AD, Zaniewski E, Anderegg N, Retention and mortality on antiretroviral therapy in sub-Saharan Africa: collaborative analyses of HIV treatment programmes. J Int AIDS Soc 2018; 21: e25084. 10.1002/jia2.25084 PMC5897849 29479867

