# Supplementary material for: Excess mortality associated with mental illness in people living with HIV in Cape Town, South Africa: a cohort study using linked electronic health records
Source: Lancet Glob Health. Author manuscript; Available in PMC 2020 Oct 23. (PMC7582785; doi:10.1016/S2214-109X(20)30279-5)
Supplement: 1 [file NIHMS1633386-supplement-1.pdf]

THE LANCET
Global Health

Supplementary appendix

This appendix formed part of the original submission and has been peer reviewed. We post it as supplied by the authors.

Supplement to: Haas AD, Ruffieux Y, van den Heuvel LL, et al. Excess mortality associated with mental illness in people living with HIV in Cape Town, South Africa: a cohort study using linked electronic health records. *Lancet Glob Health* 2020; **8:** e1326–34.

Table S1. Probability of death, loss to follow-up, retention in care with viral load suppression, and retention in care with non-suppressed viral load in patients with or without history of mental illness after 2, 4, 6, 8, and 10 years on ART

	Years on antiretroviral therapy (ART)				
	**2 years**	**4 years**	**6 years**	**8 years**	**10 years**
**Patients with history of mental illness**					
Died	8.1 (6.9- 9.2)	13.6 (12.1-15.1)	19.2 (17.3-21.0)	24.5 (22.2-26.7)	32.0 (28.9-35.1)
Lost to follow-up	20.5 (18.8-22.1)	27.0 (25.1-28.9)	31.0 (28.9-33.2)	33.9 (31.4-36.3)	34.0 (30.8-37.1)
Retained in care with VLS	49.6 (47.5-51.7)	46.2 (44.1-48.3)	40.3 (38.1-42.5)	35.7 (33.4-38.1)	28.9 (26.2-31.7)
Retained in care with NVL	21.9 (20.2-23.6)	13.2 (11.7-14.6)	9.5 (8.2-10.8)	5.9 (4.7- 7.2)	5.1 (3.5- 6.7)
**Patients without history of mental illness**					
Died	3.9 (3.7- 4.1)	6.5 (6.2- 6.7)	9.2 (8.8- 9.5)	11.3 (10.9-11.7)	13.4 (12.9-13.9)
Lost to follow-up	25.8 (25.4-26.2)	33.8 (33.4-34.3)	38.6 (38.1-39.1)	42.4 (41.8-43.0)	45.3 (44.6-46.0)
Retained in care with VLS	51.7 (51.2-52.2)	50.4 (49.9-50.9)	46.5 (46.0-47.0)	42.0 (41.5-42.6)	38.0 (37.4-38.7)
Retained in care with NVL	18.6 (18.2-19.0)	9.3 (9.0- 9.6)	5.8 (5.5- 6.0)	4.2 (4.0- 4.5)	3.2 (2.9- 3.5)

95% confidence intervals are shown in parentheses. ART, antiretroviral therapy, VLS, viral load suppression; NVL, non-suppressed viral load.

Table S2. Probability of death, loss to follow-up, retention in care with viral load suppression, and retention in care with non-suppressed viral load in patients with or without history of mental illness after 2, 4, 6, 8, and 10 years on ART by treatment program

	Years on antiretroviral therapy (ART)				
	2 years	4 years	6 years	8 years	10 years
**GUGULETHU**					
**Patients with history of mental illness**					
Died	6.2 (3.3- 9.1)	11.3 (7.1-15.5)	19.3 (13.5-25.0)	27.5 (19.9-35.1)	33.0 (24.2-41.7)
Lost to follow-up	29.2 (24.0-34.3)	35.5 (29.7-41.3)	40.1 (33.5-46.7)	41.0 (33.0-49.0)	45.9 (36.3-55.6)
Retained in care with VLS	52.7 (46.8-58.5)	41.1 (35.4-46.8)	32.0 (26.6-37.5)	27.4 (22.0-32.9)	15.2 (8.7-21.7)
Retained in care with NVL	12.0 (8.0-15.9)	12.1 (8.3-15.9)	8.6 (5.3-11.9)	4.0 (1.7- 6.4)	5.9 (1.3-10.5)
**Patients without history of mental illness**					
Died	4.4 (3.9- 4.9)	8.1 (7.4- 8.8)	11.3 (10.4-12.2)	13.8 (12.8-14.9)	15.9 (14.6-17.2)
Lost to follow-up	34.1 (33.0-35.2)	43.8 (42.6-45.0)	49.2 (48.0-50.5)	53.5 (52.1-54.9)	58.8 (57.1-60.5)
Retained in care with VLS	54.2 (53.0-55.3)	43.1 (41.9-44.2)	35.6 (34.4-36.7)	30.0 (28.8-31.2)	23.5 (22.2-24.9)
Retained in care with NVL	7.3 (6.7- 8.0)	5.0 (4.5- 5.5)	3.9 (3.4- 4.4)	2.7 (2.2- 3.1)	1.7 (1.1- 2.3)
**KHAYELITSHA**					
**Patients with history of mental illness**					
Died	7.1 (5.8- 8.4)	11.9 (10.2-13.6)	15.9 (13.9-17.9)	19.1 (16.7-21.4)	23.1 (20.2-26.0)
Lost to follow-up	18.7 (16.8-20.6)	25.2 (23.0-27.5)	30.0 (27.5-32.5)	35.1 (32.3-37.9)	37.7 (34.4-41.1)
Retained in care with VLS	48.5 (46.1-51.0)	48.4 (45.8-50.9)	43.5 (40.9-46.2)	38.5 (35.6-41.5)	33.2 (29.8-36.6)
Retained in care with NVL	25.7 (23.5-27.9)	14.5 (12.7-16.3)	10.5 (8.8-12.2)	7.3 (5.6- 9.0)	5.9 (3.9- 8.0)
**Patients without history of mental illness**					
Died	3.4 (3.2- 3.6)	5.4 (5.2- 5.7)	7.6 (7.2- 7.9)	9.1 (8.7- 9.5)	10.7 (10.2-11.2)
Lost to follow-up	24.2 (23.7-24.7)	31.7 (31.2-32.3)	36.7 (36.1-37.2)	40.7 (40.1-41.4)	43.4 (42.7-44.1)
Retained in care with VLS	50.8 (50.3-51.4)	52.5 (51.9-53.1)	49.6 (49.0-50.2)	45.6 (45.0-46.3)	42.4 (41.6-43.1)
Retained in care with NVL	21.6 (21.1-22.1)	10.4 (10.0-10.7)	6.2 (5.9- 6.5)	4.6 (4.2- 4.9)	3.6 (3.2- 3.9)
**TYGERBERG**					
**Patients with history of mental illness**					
Died	13.9 (10.2-17.6)	21.8 (17.4-26.2)	30.4 (25.6-35.3)	38.9 (33.4-44.5)	52.7 (45.8-59.5)
Lost to follow-up	21.1 (16.8-25.3)	28.1 (23.4-32.7)	28.7 (23.9-33.4)	26.5 (21.6-31.5)	21.0 (14.6-27.4)
Retained in care with VLS	52.2 (47.0-57.4)	41.4 (36.3-46.5)	34.8 (29.9-39.7)	31.2 (26.2-36.2)	24.7 (18.9-30.5)
Retained in care with NVL	12.9 (9.5-16.3)	8.8 (6.0-11.6)	6.1 (3.8- 8.5)	3.3 (1.3- 5.3)	1.6 (-0.5- 3.8)
**Patients without history of mental illness**					
Died	10.2 (8.8-11.6)	16.4 (14.6-18.1)	23.6 (21.6-25.6)	29.7 (27.5-31.9)	35.8 (33.2-38.4)
Lost to follow-up	22.3 (20.5-24.2)	29.3 (27.2-31.4)	29.5 (27.4-31.6)	29.6 (27.3-31.8)	29.6 (26.9-32.3)
Retained in care with VLS	58.1 (55.8-60.3)	48.0 (45.7-50.4)	41.9 (39.6-44.3)	35.8 (33.4-38.2)	31.3 (28.5-34.1)
Retained in care with NVL	9.4 (8.1-10.7)	6.3 (5.2- 7.4)	5.0 (4.0- 5.9)	4.9 (3.7- 6.1)	3.3 (1.8- 4.8)

95% confidence intervals are shown in parentheses. ART, antiretroviral therapy, VLS, viral load suppression; NVL, non-suppressed viral load.

Table S3. Factors associated with mortality after initiation of antiretroviral therapy

	All-cause mortality[1]				Mortality from natural cause[2]				Mortality from unnatural cause[3]			
	Univariable	Model 1	Model 2	Model 3	Univariable	Model 1	Model 2	Model 3	Univariable	Model 1	Model 2	Model 3
	HR (95% CI)	aHR (95% CI)	aHR (95% CI)	aHR (95% CI)	aHR (95% CI)	aHR (95% CI)	aHR (95% CI)	aHR (95% CI)	aHR (95% CI)	aHR (95% CI)	aHR (95% CI)	aHR (95% CI)
History of mental illness												
No	1.00	1.00	1.00	1.00	1.00	1.00	1.00	1.00	1.00	1.00	1.00	1.00
Yes	3.05 (2.75-3.37)	2.98 (2.69-3.30)	2.76 (2.50-3.06)	2.73 (2.46-3.02)	3.14 (2.81-3.51)	3.00 (2.69-3.36)	2.78 (2.48-3.11)	2.75 (2.46-3.08)	2.15 (1.29-3.56)	2.10 (1.27-3.49)	2.05 (1.23-3.41)	2.07 (1.24-3.44)
Sex												
Male	1.00	1.00	1.00	1.00	1.00	1.00	1.00	1.00	1.00	1.00	1.00	1.00
Female	0.54 (0.51-0.58)	0.61 (0.57-0.65)	0.71 (0.67-0.76)	0.75 (0.70-0.80)	0.58 (0.54-0.62)	0.66 (0.61-0.71)	0.77 (0.72-0.83)	0.81 (0.75-0.88)	0.30 (0.23-0.39)	0.31 (0.23-0.40)	0.32 (0.24-0.42)	0.32 (0.24-0.43)
Age, year												
15-24	1.00	1.00	1.00	1.00	1.00	1.00	1.00	1.00	1.00	1.00	1.00	1.00
25-34	1.33 (1.12-1.58)	1.19 (1.01-1.41)	1.05 (0.89-1.25)	1.12 (0.95-1.33)	1.40 (1.15-1.71)	1.28 (1.05-1.56)	1.13 (0.92-1.37)	1.21 (0.99-1.48)	1.38 (0.63-3.03)	1.11 (0.51-2.45)	1.06 (0.48-2.33)	1.08 (0.49-2.38)
35-44	1.71 (1.44-2.03)	1.40 (1.18-1.66)	1.15 (0.97-1.37)	1.31 (1.10-1.56)	1.81 (1.49-2.21)	1.54 (1.26-1.88)	1.26 (1.03-1.54)	1.45 (1.19-1.78)	2.01 (0.92-4.38)	1.29 (0.58-2.84)	1.20 (0.54-2.66)	1.24 (0.56-2.76)
45-54	2.41 (2.02-2.87)	1.87 (1.56-2.23)	1.53 (1.28-1.83)	1.78 (1.48-2.13)	2.59 (2.11-3.17)	2.09 (1.70-2.58)	1.69 (1.37-2.09)	2.02 (1.64-2.49)	2.14 (0.95-4.83)	1.23 (0.54-2.82)	1.14 (0.49-2.61)	1.18 (0.51-2.72)
55-64	4.02 (3.32-4.87)	3.02 (2.49-3.68)	2.48 (2.04-3.02)	2.87 (2.36-3.49)	4.46 (3.58-5.56)	3.49 (2.79-4.37)	2.83 (2.26-3.54)	3.35 (2.67-4.20)	1.60 (0.57-4.46)	0.88 (0.31-2.47)	0.81 (0.29-2.29)	0.84 (0.29-2.37)
65+	5.95 (4.55-7.79)	4.54 (3.46-5.96)	3.75 (2.86-4.92)	4.26 (3.25-5.59)	7.10 (5.28-9.55)	5.65 (4.19-7.62)	4.61 (3.42-6.22)	5.33 (3.95-7.20)	2.62 (0.54-12.69)	1.39 (0.28-6.80)	1.29 (0.26-6.33)	1.30 (0.27-6.39)
Year of ART initiation												
2004-2007	1.00	1.00	1.00	1.00	1.00	1.00	1.00	1.00	1.00	1.00	1.00	1.00
2008-2011	1.22 (1.10-1.36)	1.15 (1.04-1.28)	1.29 (1.16-1.43)	1.18 (1.06-1.31)	1.38 (1.23-1.56)	1.29 (1.15-1.46)	1.45 (1.28-1.63)	1.29 (1.14-1.45)	1.03 (0.67-1.59)	1.01 (0.65-1.55)	1.02 (0.66-1.57)	0.98 (0.63-1.52)
2012-2014	1.11 (0.98-1.25)	1.03 (0.91-1.17)	1.41 (1.24-1.60)	1.24 (1.09-1.41)	1.38 (1.19-1.59)	1.27 (1.10-1.47)	1.76 (1.52-2.04)	1.47 (1.27-1.70)	1.48 (0.89-2.44)	1.46 (0.88-2.42)	1.53 (0.91-2.56)	1.42 (0.85-2.39)
2015-2017	0.91 (0.77-1.07)	0.82 (0.70-0.96)	1.32 (1.12-1.56)	1.23 (1.04-1.45)	1.15 (0.96-1.38)	1.03 (0.86-1.24)	1.69 (1.40-2.04)	1.50 (1.24-1.81)	1.26 (0.62-2.53)	1.25 (0.62-2.53)	1.29 (0.63-2.67)	1.17 (0.56-2.42)
CD4 cell count per µL												
<100	1.00		1.00	1.00	1.00		1.00	1.00	1.00		1.00	1.00
100-199	0.59 (0.55-0.64)		0.71 (0.66-0.78)	0.72 (0.67-0.79)	0.59 (0.54-0.64)		0.71 (0.65-0.78)	0.72 (0.66-0.79)	0.88 (0.60-1.30)		1.03 (0.69-1.53)	1.03 (0.69-1.53)
200-349	0.38 (0.34-0.42)		0.49 (0.44-0.54)	0.50 (0.46-0.56)	0.37 (0.33-0.41)		0.47 (0.42-0.52)	0.49 (0.44-0.55)	0.86 (0.57-1.30)		1.02 (0.67-1.56)	1.02 (0.67-1.56)
350-499	0.38 (0.32-0.45)		0.49 (0.41-0.59)	0.50 (0.42-0.59)	0.37 (0.30-0.44)		0.46 (0.38-0.56)	0.46 (0.38-0.57)	1.27 (0.71-2.30)		1.58 (0.86-2.90)	1.55 (0.85-2.85)
500+	0.31 (0.24-0.39)		0.43 (0.34-0.54)	0.43 (0.34-0.55)	0.32 (0.25-0.42)		0.43 (0.33-0.56)	0.43 (0.33-0.56)	0.43 (0.13-1.40)		0.61 (0.19-1.98)	0.59 (0.18-1.92)
Missing	0.57 (0.52-0.62)		0.67 (0.61-0.74)	0.64 (0.58-0.70)	0.56 (0.51-0.62)		0.66 (0.59-0.73)	0.62 (0.56-0.69)	1.09 (0.73-1.64)		1.24 (0.82-1.87)	1.25 (0.83-1.88)
WHO clinical stage												
1	1.00		1.00	1.00	1.00		1.00	1.00	1.00		1.00	1.00
2	1.40 (1.24-1.57)		1.22 (1.08-1.37)	1.23 (1.09-1.39)	1.39 (1.22-1.59)		1.23 (1.07-1.40)	1.25 (1.09-1.43)	1.88 (1.21-2.93)		1.68 (1.08-2.63)	1.70 (1.09-2.66)
3	2.45 (2.23-2.70)		1.87 (1.69-2.06)	1.81 (1.63-2.00)	2.45 (2.20-2.73)		1.91 (1.71-2.14)	1.85 (1.65-2.07)	1.82 (1.22-2.72)		1.46 (0.96-2.22)	1.46 (0.96-2.23)
4	3.54 (3.19-3.93)		2.44 (2.19-2.73)	2.40 (2.15-2.68)	3.55 (3.16-4.00)		2.52 (2.23-2.85)	2.47 (2.18-2.79)	2.09 (1.33-3.29)		1.60 (0.99-2.58)	1.59 (0.98-2.58)
Missing	1.42 (1.16-1.74)		1.37 (1.12-1.68)	1.27 (1.04-1.56)	1.40 (1.11-1.75)		1.37 (1.09-1.72)	1.26 (1.00-1.58)	1.59 (0.73-3.44)		1.58 (0.72-3.44)	1.55 (0.71-3.38)
HIV treatment outcome												
Retained in care with VLS	1.00			1.00	1.00			1.00	1.00			1.00
Retained in care with NVL	3.04 (2.78-3.33)			2.97 (2.72-3.25)	3.43 (3.09-3.81)			3.38 (3.04-3.75)	0.83 (0.54-1.27)			0.79 (0.52-1.22)
LTFU	2.35 (2.17-2.54)			2.41 (2.22-2.61)	2.90 (2.64-3.17)			2.97 (2.70-3.25)	1.41 (1.05-1.91)			1.34 (0.99-1.82)

[1] Unadjusted and adjusted hazard ratios with 95% confidence intervals.

[2] Unadjusted and adjusted cause-specific hazard ratios hazard ratios with 95% confidence intervals.

Analyses were adjusted for all variables shown in the model and antiretroviral therapy program. CD4 cell count, and WHO clinical stage were assessed at initiation of antiretroviral therapy.

History of mental illness, age, and HIV treatment outcome were modeled as time-varying variables. HR, hazard ratio; aHR, adjusted hazard ratio; CI, confidence interval; ART, antiretroviral therapy; WHO, World Health Organization; VLS, viral load suppression; NVL, non-suppressed viral load; LTFU, loss to follow-up.

Table S4: Adjusted hazard ratios for all-cause mortality after ART initiation in patients with mental illness for various definitions of the exposure variable

	Univariable analysis	Multivariable analysis
Definition	HR (95% CI)	aHR (95% CI)
Exposed for 1 year after each mental health treatment		
Unexposed	1.00	1.00
Exposed	3.66 (3.24-4.13)	3.49 (3.09-3.94)
Exposed for 2 years after each mental health treatment		
Unexposed	1.00	1.00
Exposed	3.41 (3.05-3.81)	3.21 (2.87-3.59)
Exposed for 3 years after each mental health treatment		
Unexposed	1.00	1.00
Exposed	3.36 (3.02-3.73)	3.15 (2.83-3.50)
Exposed for 4 years after each mental health treatment		
Unexposed	1.00	1.00
Exposed	3.23 (2.91-3.59)	3.01 (2.71-3.35)
Exposed for 5 years after each mental health treatment		
Unexposed	1.00	1.00
Exposed	3.12 (2.81-3.46)	2.90 (2.61-3.22)

Data are hazard ratios (HR) and adjusted hazard ratios (aHR) with 95% confidence intervals (CI). Multivariable analyses were adjusted for age, sex, year of ART initiation, CD4, and WHO clinical stage at ART initiation and treatment program.

Mental illness was modeled as a time-varying binary variable. Patients were considered unexposed to mental illness until they received the first mental health treatment; thereafter patients were considered to be affected by mental illness (exposed) for 1, 2, 3, 4, and 5 years after each mental health treatment (i.e. dispensing of psychiatric medication or hospital admission for mental disorder) and unexposed thereafter. Patients who never received mental health treatment were considered unexposed throughout follow-up.

Figure S1. HIV treatment outcomes and all-cause mortality during 10 years of antiretroviral therapy for patients with and without history of mental illness by treatment program

Percentage of patients who were retained in care with non-suppressed viral load, retained in care with viral load suppression, lost to follow-up, or had died over 10 years of antiretroviral therapy for patients who initiated ART at Gugulethu (A), Khayelitsha (B), or Tygerberg (C) and with or without history of mental illness.

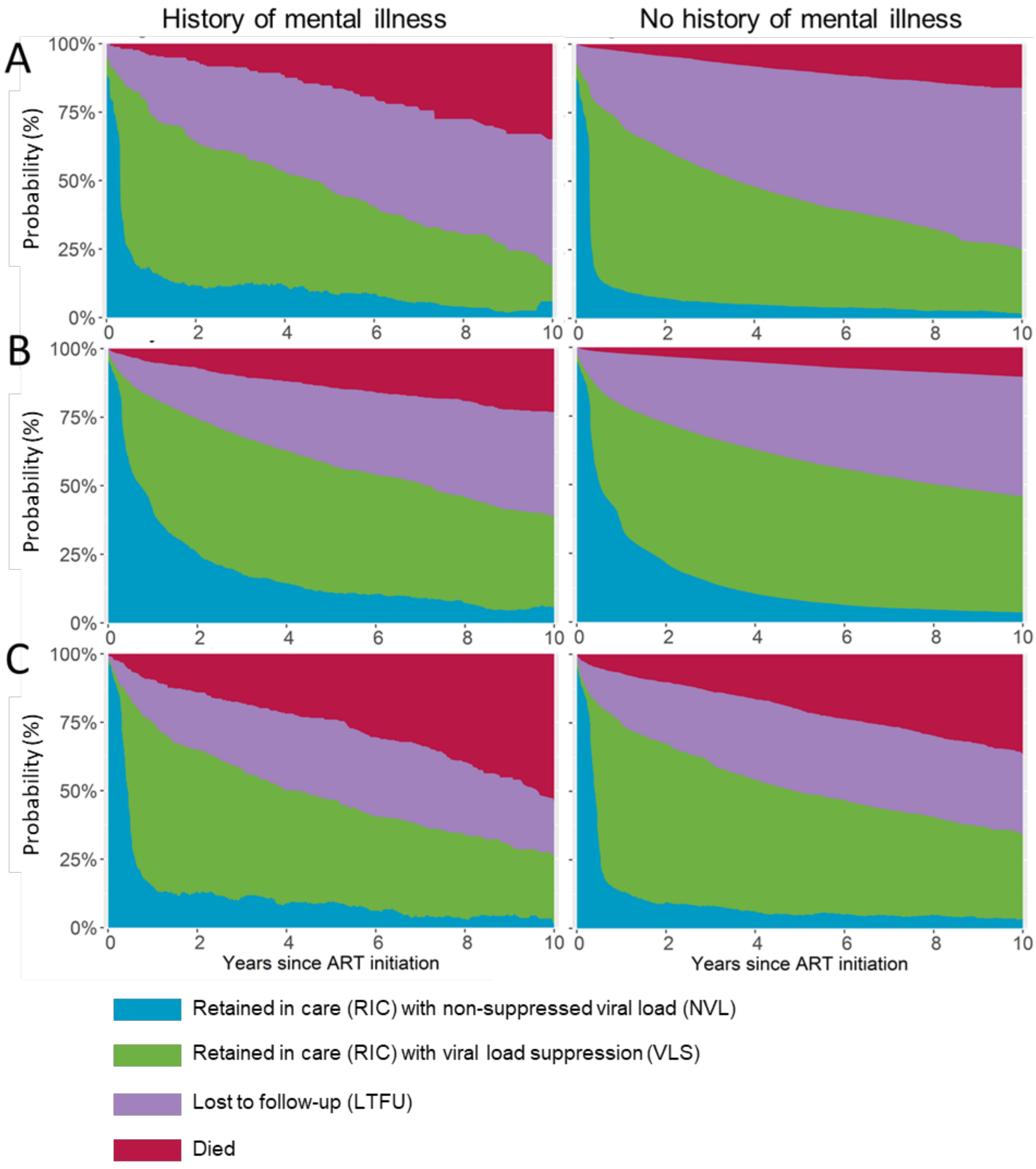

Figure S2. Adjusted hazard ratios comparing mortality and HIV treatment outcomes between patients who received mental health treatment during antiretroviral therapy and patients without history of mental illness

Adjusted hazard ratios and 95% confidence intervals (in parentheses) comparing transition rates for patients who received mental health treatment during antiretroviral therapy (n=2445) and patients without history of mental illness for each transition of the multi-state model. Patients without history of mental illness were the reference group. The width of the arrow is proportional to the strength of the association. Hazard ratios were adjusted for age, sex, year of ART initiation, CD4 cell count, WHO clinical stage at ART initiation, cumulative time with NVL, and treatment program. Values with 95% confidence intervals that do not include 1 are shown in bold.

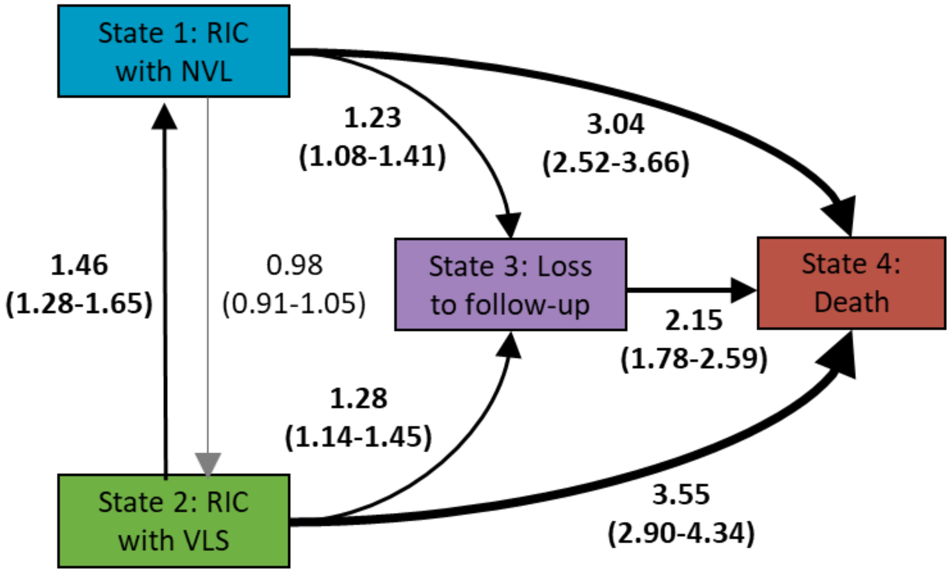

RIC, retained in care; NVL, non-suppressed viral load; VLS, viral load suppression.

Figure S3. Adjusted hazard ratios comparing mortality and HIV treatment outcomes between patients who received mental health treatment before but not after initiation of antiretroviral therapy and patients without history of mental illness

Adjusted hazard ratios and 95% confidence intervals (in parentheses) comparing transition rates for patients who received mental health treatment before but not after initiation of antiretroviral therapy (n=482) and patients without history of mental illness for each transition of the multi-state model. Patients without history of mental illness were the reference group. The width of the arrow is proportional to the strength of the association. Hazard ratios were adjusted for age, sex, year of ART initiation, CD4 cell count, WHO clinical stage at ART initiation, cumulative time with NVL, and treatment program. Values with 95% confidence intervals that do not include 1 are shown in bold.

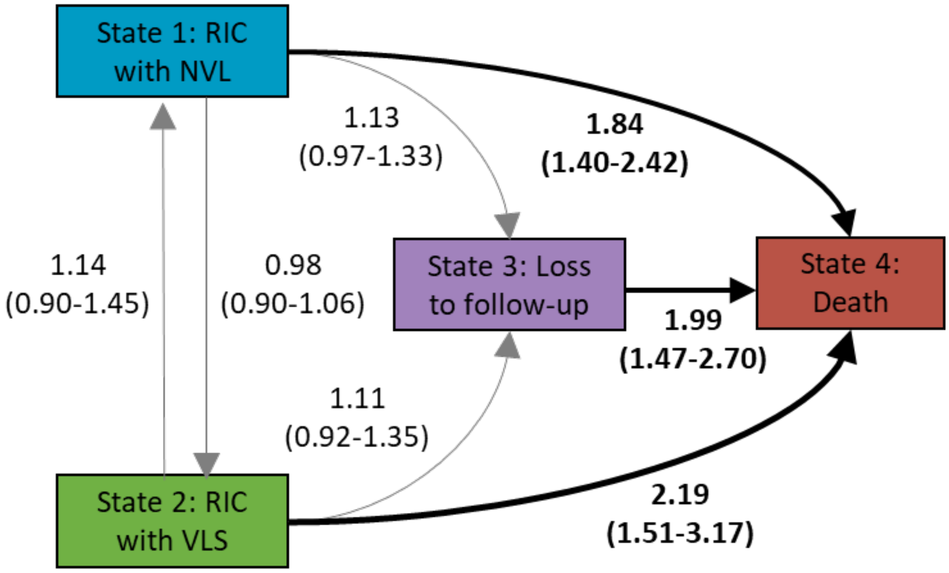

RIC, retained in care; NVL, non-suppressed viral load; VLS, viral load suppression.

Figure S4. Adjusted hazard ratios comparing mortality and HIV treatment outcomes between patients who had been hospitalized for a mental disorder and patients without history of mental illness

Adjusted hazard ratios and 95% confidence intervals (in parentheses) comparing transition rates for patients who had ever been hospitalized for a mental disorder (n=923) and patients without history of mental illness (n=55 737) for each transition of the multi-state model. Patients without history of mental illness were the reference group. The width of the arrow is proportional to the strength of the association. Hazard ratios were adjusted for age, sex, year of ART initiation, CD4 cell count, WHO clinical stage at ART initiation, cumulative time with NVL, and treatment program. Values with 95% confidence intervals that do not include 1 are shown in bold.

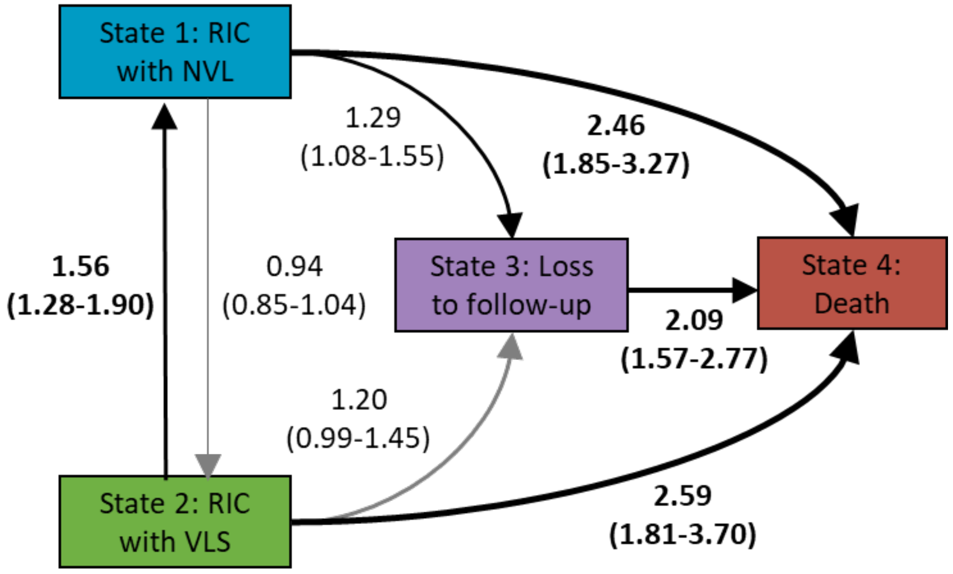

RIC, retained in care; NVL, non-suppressed viral load; VLS, viral load suppression.

Figure S5. Adjusted hazard ratios comparing mortality and HIV treatment outcomes between patients who had received psychiatric medication but had never been hospitalized for a mental illness and patients without history of mental illness

Adjusted hazard ratios and 95% confidence intervals (in parentheses) comparing transition rates for patients who had received psychiatric medication but had never been hospitalized for a mental illness (n=1970) and patients without history of mental illness for each transition of the multi-state model. The width of the arrow is proportional to the strength of the association. Hazard ratios were adjusted for age, sex, year of ART initiation, CD4 cell count, WHO clinical stage at ART initiation, cumulative time with NVL, and treatment program. Values with 95% confidence intervals that do not include 1 are shown in bold.

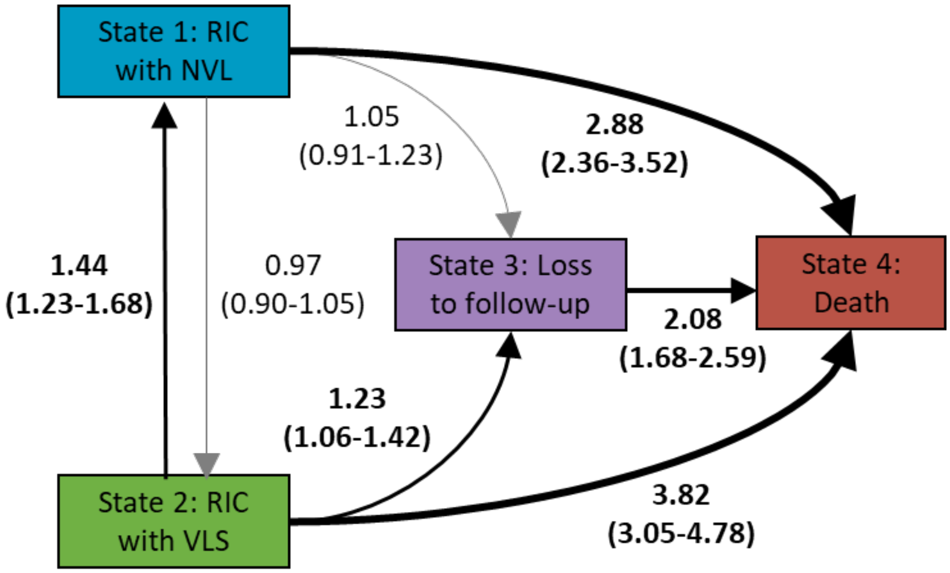

RIC, retained in care; NVL, non-suppressed viral load; VLS, viral load suppression.

Figure S6. Adjusted hazard ratios comparing mortality and HIV treatment outcomes between patients who received and did not receive antipsychotics, antidepressants, or anxiolytic medication

Adjusted hazard ratios and 95% confidence intervals (in parentheses) comparing transition rates for patients who ever received antipsychotics (n=1561), antidepressants (n=1159), or anxiolytics (n=815) for each transition of the multi-state model. Patients who never received antipsychotics, antidepressants, or anxiolytic medication were the reference group. The width of the arrow is proportional to the strength of the association. Hazard ratios were adjusted for other psychiatric medication (e.g., aHRs for antipsychotics were adjusted for use of antidepressants and anxiolytics) age, sex, year of ART initiation, CD4 cell count, WHO clinical stage at ART initiation, cumulative time with NVL, and treatment program. Values with 95% confidence intervals that do not include 1 are shown in bold.

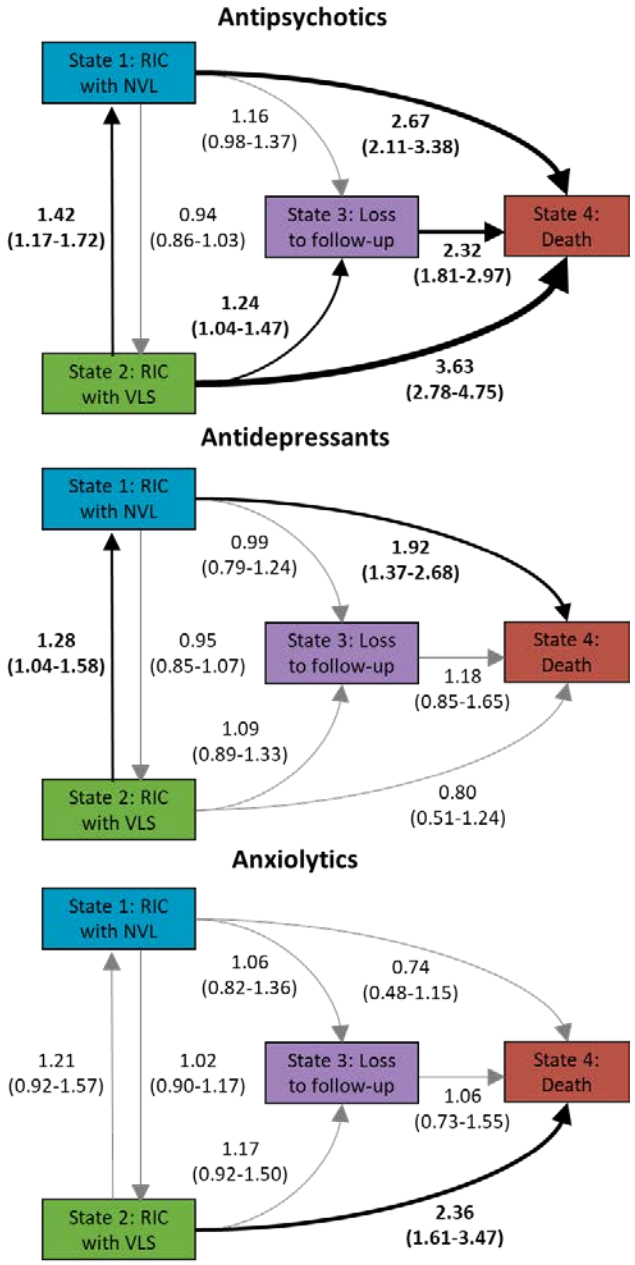

RIC, retained in care; NVL, non-suppressed viral load; VLS, viral load suppression.

Figure S7. Adjusted hazard ratios comparing mortality and HIV treatment outcomes between patients who received a psychiatric ICD10 diagnosis and patients without history of mental illness

Adjusted hazard ratios and 95% confidence intervals (in parentheses) comparing transition rates for patients who received an ICD10 diagnosis for a substance use disorder (n=103), psychotic disorder (n=182), affective disorder (n=150), or anxiety disorder (n=40) and patients without history of mental illness for each transition of the multi-state model. Patients without history of mental disorders were the reference group. The width of the arrow is proportional to the strength of the association. Hazard ratios were adjusted for age, sex, year of ART initiation, CD4 cell count, WHO clinical stage at ART initiation, cumulative time with NVL, and treatment program. Values with 95% confidence intervals that do not include 1 are shown in bold.

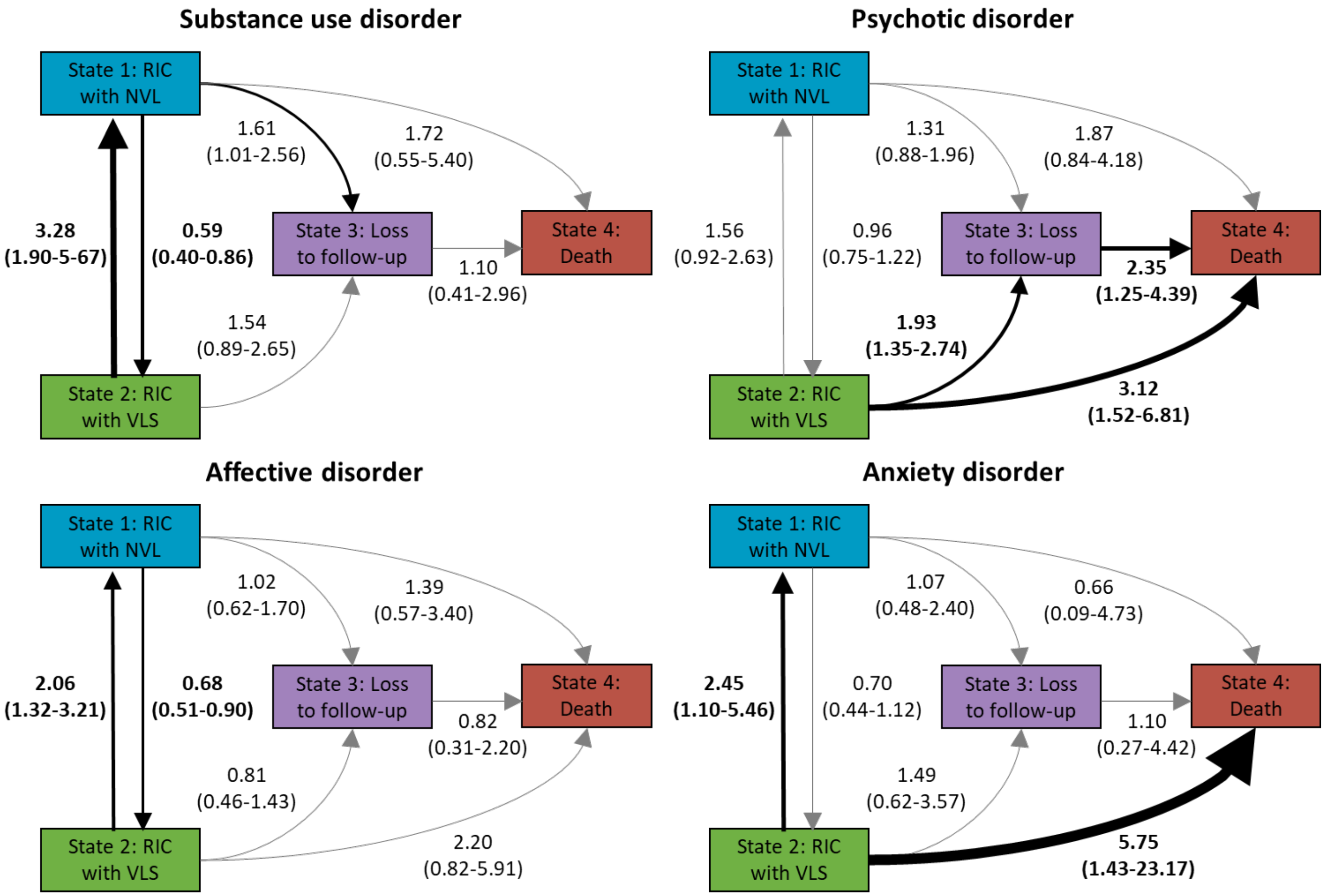

RIC, retained in care; NVL, non-suppressed viral load; VLS, viral load suppression
